# Refugees and the post-migration environment

**DOI:** 10.1186/s12916-018-1155-y

**Published:** 2018-09-04

**Authors:** Mina Fazel

**Affiliations:** Department of Psychiatry, University of Oxford, Warneford Lane, Oxford, OX3 7JX UK

**Keywords:** Refugee, Resettlement, Mental health, Parenting, Child, Adolescent, Cohort

## Abstract

The ever-increasing number of reasons forcing people to flee from their homes to new, safer places either within their countries of origin, into neighbouring countries or across continental, conversant and cultural boundaries has led to a humanitarian crisis to which scientific enquiry must increasingly contribute. Yet, little is known about how best to support refugee adults and children in the process of resettling in high-income nations, an issue which the recent study by Lau et al. published in *this journal*, is attempting to address. Their study highlights how refugee parents, children and adolescents report good child mental health and adjustment approximately 3–4 years after gaining humanitarian visa status to remain in Australia. Herein, the need to support parenting capability and to facilitate public policy to work within an evidence-based framework are discussed.

Please see related article: https://bmcmedicine.biomedcentral.com/articles/10.1186/s12916-018-1124-5.

## Background

A small proportion of the total number of refugees worldwide are currently living in high-income nations either as part of national or international resettlement programmes or having travelled using a range of either authorised or proscribed means. For these few, determining how best to facilitate adjustment to their new environment is likely one of the easier tasks to address relative to the numerous economic, humanitarian and political complexities in low- and middle-income contexts where the majority of global refugees reside.

In understanding and supporting the mental health needs of refugee populations, it is increasingly evident that two main factors need to be considered. First, the impact of the extent and number of exposures to potentially traumatic events and stressors, predominantly pre- and peri-migration, which individuals and families have experienced. Second, the influence of the post-migration environment and how it can either mitigate or accentuate any mental health difficulties [1, 2]. Therefore, how to determine the relative influence of and relationship between the multitude of potential resettlement factors on the mental health of refugee populations in the post-migration environment is a key issue to explore so that interventions can potentially be tailored to facilitate components of this process [3].

## Building a new life in Australia

Lau et al. [4] report on the Building a New Life in Australia (BNLA) study, a large longitudinal prospective cohort of Australian refugees, recruited within 6 months of being granted a humanitarian visa to remain. The study includes a representative refugee sample collected from 11 different sites across Australia, comprised of refugees from different cultural backgrounds, with a range of visa types granted, and living in both urban and regional resettlement environments.

The relative dearth of longitudinal studies on resettled refugees has been a limiting feature of this discipline, exacerbated by a number of cultural and contextual factors that can make research difficult for this relatively mobile population [2]. The BNLA study is thus timely and important as it plans to examine the cohort at five time-points and adopts an ecological framework to inform components of its investigation. The data presented by Lau et al. [4] was obtained from the third study wave, at 2–3 years post-recruitment, when field workers conducted interviews during home visits, mostly in native languages. The data reported was from 426 adults who identified as primary caregivers of children (from a sample of almost 2000 adults) and 694 children and adolescents, with adolescents preferentially sampled so as to maximise those that could complete self-report measures [4]. The study obtained impressive findings, including both parents and adolescents reporting that children and adolescents were adjusting well in their new environment and, in some areas, with better adjustment than expected compared to age-matched Australian norms. Further, high levels of parent- and self-reported academic achievement, physical activity in boys, and involvement in extracurricular activities were reported, although increased peer problems at younger ages and an increase in emotional problems for older adolescent girls were also observed according to Strengths and Difficulties Questionnaire data [4].

There are two areas, of the many different findings, that are important to highlight. First is the welcome focus on the parental role and some of the difficulties that can be encountered for refugee parents moving to a new environment. These difficulties lie in the needs to support their own mental health, the challenges of adapting to a new parental role in a different cultural context to which their children might adjust and adopt more readily, and finally being able to provide for the socioeconomic necessities of their family [2]. Given how crucial parenting is, finding ways to prevent mental health problems cascading across generations and to address the cluster of adversities that can affect family wellbeing is likely to be an important arena of intervention. This study asked parents about their parenting and reported how a measure of harshness was associated with poorer child outcomes. Difficulties in parenting in the post-migration context have been reported in both qualitative and quantitative studies with, for example, economic hardship forcing adaptation strategies that impair positive parent–child interactions as well as parental psychological distress contributing to harsh parenting [5]. There have been intervention studies to support refugee families, for example, by demonstrating improvements in positive-parenting practices [2, 6]; these need to be further conceptualised and developed.

Second, is the importance of developing a robust evidence-base to inform and influence public policy, especially for refugees and victims of organised violence, where it seems that many governments are adopting harsher social policies that can prove detrimental to mental health. The study by Lau et al. [4] reports on a group who have been relatively supported in their immigration journey, having been granted a humanitarian visa, often within their first year in Australia. Their adjustment outcomes, as reported in the BNLA study, are highly positive, mirroring other studies where forced migrants, when they feel safe and supported in the post-migration environment, have high functional outcomes [7]. Additionally, the BNLA study was undertaken by both the Australian Government Department of Social Services and the Australian Institute of Family Studies, showing that ensuring a collaborative approach and trying to embed such studies, at the outset, within the relevant government agencies will increase the likelihood that any findings of relevance to public policy are appreciated, disseminated and implemented early on by the pertinent agencies.

## Conclusions

Lau et al.’s study [4] represents an important shift in the assessment of the mental health of forcibly displaced populations. The authors assessed the post-migration influences on outcomes and will hopefully inspire further studies to encapsulate more of the myriad and multifaceted components that can mitigate risks and support mental health and successful resettlement [8]. Supplementary studies that start as early as possible in the migration journey and include all possible migration routes and outcomes to reflect the scope of forced migration experiences are needed. Increased utilisation of mixed methods of enquiry and including involvement of the refugee voice in the hypotheses and process of investigation will continue to enhance our understanding of this field. How host-population interventions might support refugees remains unknown; however, there is reason for optimism as resettlement outcomes for communities can be enhanced. It is a human rights imperative that this is now realised.
